# Plasma exosomes lncRNA-miRNA-mRNA network construction and its diagnostic efficacy identification in first-episode schizophrenia

**DOI:** 10.1186/s12888-023-05052-9

**Published:** 2023-08-21

**Authors:** Xinzhe Du, Jinzhi Lv, Jianping Feng, Xinrong Li, Yao Gao, Xiao Wang, Wentao Zhao, Zhiyong Ren, Ruifang Zhang, Xiaohua Cao, Sha Liu, Yong Xu

**Affiliations:** 1 Department of Psychiatry, First Hospital/First Clinical Medical College of Shanxi Medical University, Taiyuan, China; 2 Shanxi Key Laboratory of Artificial Intelligence Assisted Diagnosis and Treatment for Mental Disorder, First Hospital of Shanxi Medical University, Taiyuan, China; 3 Department of Physiology, Shanxi Medical University, Taiyuan, China; 4 Female Department of Schizophrenia, Shanxi Province Mental Health Center/Taiyuan Psychiatric Hospital, Taiyuan, China; 5 Department of Psychiatry, Taiyuan Central Hospital of Shanxi Medical University, Taiyuan, Shanxi Province China

**Keywords:** Schizophrenia, Exosome, lncRNA, miRNA, mRNA

## Abstract

**Background:**

The exosomal lncRNA-miRNA-mRNA networks in first episode schizophrenia (FOS) have not reported yet. This study examined the lncRNA, miRNA and mRNA expression level in exosome derived from first episode schizophrenia (FOS) patients, and explored the the potential of exosomes as biomarkers for schizophrenia.

**Methods:**

We recruited 10 FOS patients and healthy controls (HCs) respectively, examined the lncRNA, miRNA and mRNA expression level of plasma exosome by high throughput sequencing, constructed lncRNA-miRNA-mRNA network, and performed correlation analysis, GO and KEGG pathway analysis, PPI network construction and ROC analysis.

**Results:**

There were 746 differently expressed lncRNA, 22 differently expressed miRNA, and 2637 differently expressed mRNA in plasma exosome in FOS compared with HCs. Then we constructed ceRNA network consisting of 8 down-regulated lncRNA, 7 up-regulated miRNA and 65 down-regulated mRNA, and 1 up-regulated lncRNA, 1 down-regulated miRNA and 4 up-regulated mRNA. The expression level of 1 lncRNA and 7 mRNA in exosomal network were correlated with PANSS score. GO and KEGG pathway analysis showed that 4 up-regulated mRNAs were enriched in neuropsychiatric system function. Down-regulated mRNA EZH2 and SIRT1 were identified as hub gene. Finally, we detected the ROC curve of ENSG00000251562, miR-26a-5p, EZH2, miR-22-3p, SIRT1, ENSG00000251562—miR-26a-5p—EZH2, ENSG00000251562—miR-22-3p—SIRT1, and found that the AUC of ceRNA network was higher than lncRNA, miRNA and mRNA alone.

**Conclusion:**

We constructed the lncRNA-miRNA-mRNA network in exosome derived from FOS plasma, and found that lncRNA-miRNA-mRNA network has potential as biomarkers for FOS.

**Supplementary Information:**

The online version contains supplementary material available at 10.1186/s12888-023-05052-9.

## Introduction

Schizophrenia (SZ) is one of the most common severe mental disorders in clinic, with a lifetime prevalence rate of about 1%. About 5–6% of patients with SZ die of suicide, and their life expectancy is reduced by about 10–20 years worldwide [1]. The 2019 Chinese Mental Health Survey showed that the lifetime prevalence of SZ in China has been increasing in recent years [2]. SZ often occurs in adolescence and early adulthood, is a disease with high recurrence rate and disability rate, easily leading to mental decline and disability [3], which brings heavy burden to patients, their families and even the whole society. At present, the diagnosis of SZ is still mainly based on clinical interviews and observations. These description methods are subjective and variable, which may lead to delayed diagnosis or misdiagnosis. Therefore, it is meaningful to find more objective diagnosis methods such as biomarkers for effective treatment of SZ.

Biomarkers refer to indicators that play an indicative role in biological responses, pathological processes, or pharmacological responses to therapeutic interventions, and have the characteristics of objective detection and evaluation. Message RNA (mRNA) and non-coding RNA in peripheral blood has been reported as a biomarker of SZ [4]. The mRNA, selenium-binding protein 1 (SELENBP1) and mitochondrial complex I, and the microRNA (miRNA) has-miR-34a and has-miR-432, were potentially useful biomarker for SZ [5]. Long non-coding RNAs (lncRNAs) showed aberrant expression in SZ [6] and some lncRNA, such as XIST, could be regarded as candidates for biomarker [7]. LncRNAs could act as endogenous molecular sponges of miRNAs to indirectly regulate mRNA expression, which is also known as ceRNA (competitive endogenous RNA) network [8]. In recent years, many studies have shown that dysregulation of the lncRNA-miRNA-mRNA network was related to the pathogenesis of many diseases such as cancers [9]. Nevertheless, the potential roles of lncRNA-miRNA-mRNA in SZ has not been explored yet.

Exosomes are membrane vesicles with a diameter of 30–150 nm, wrapped with protein and nucleic acid (mRNA, miRNA, lncRNA, etc.) and other biomolecules. Neurons, microglias, astrocytes, etc., can secrete exosomes and absorb exosomes secreted by other cells [10] Exosomes secreted by nerve cells are involved in a variety of physiological processes, including synaptic plasticity, nerve regeneration, and inflammatory responses, and played an important role in neurodegenerative diseases, such as Alzheimer's disease and Parkinson's disease [11, 12]. Recently many studies suggestetd that exosomes are promising biomarkers for SZ and depression [13, 14]. However, lncRNA-miRNA-mRNA in SZ exosomes has not been reported.

**Fig. 1:**
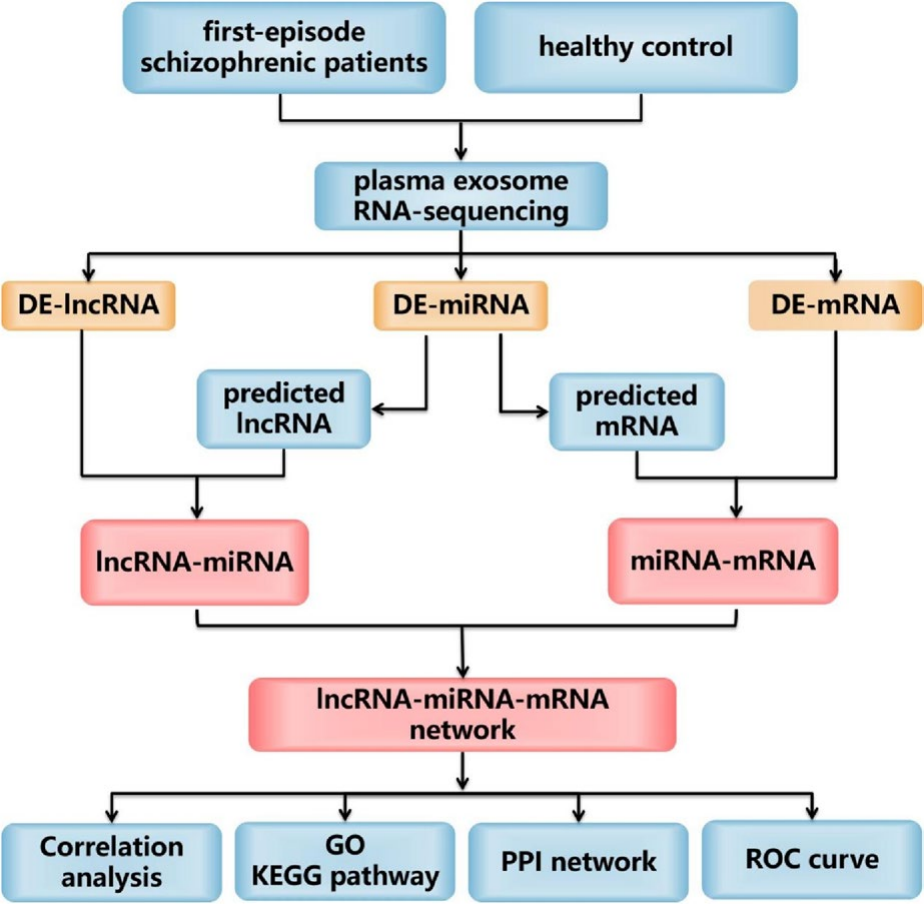
The flowchart of this study

In this study, we used high-throughput sequencing to detect the expression levels of lncRNA, miRNA and mRNA in plasma exosomes of HCs and FOS patients, and combined with bioinformatics methods to construct lncRNA-miRNA-mRNA networks, correlation analysis of lncRNAs with PANSS score, miRNAs and mRNAs in network, GO and KEGG pathway and PPI network of mRNAs in network were performed. Finally, the sensitivity of lncRNA-miRNA-mRNA networks as diagnostic markers was identified to provide new ideas for the diagnosis of diseases (Fig. 1).

## Methods

### Subjects

Ten first episode schizophrenic (FOS) were recruited from First Hospital of Shanxi Medical University according to the following criteria:1. diagnosed as SZ according to the Diagnostic and Statistical Manual of Mental Disorders Fifth Edition (DSM-5), 2. the first episode of SZ and no history of drug treatment, 3.The han nationality, 4. the score of PNASS ≥ 60. 10 healthy controls (HCs) were recruited from the community. They were selected to match to the FOS for gender, age and years of education. Those who had any prior medial diagnosis, neuropsychiatric disorders, substance abuse were excluded.

This study was approved by the Research Ethics Committee of the First Hospital of Shanxi Medical University, and written informed consents were obtained from all participants. All methods were performed in accordance with the relevant guidelines and regulations.

### Exosome isolation

About 5 ml of plasma was isolated from 10 ml of peripheral blood. The plasma exosome was extracted from 5 ml plasma of participants. The exosomes were isolated using the exoEasy Maxi Kit (Qiagen) following the manufacturer’s protocol.

### RNA isolation and RNA sequencing

Exosomes performed RNA sequencing at the Cloud-Seq Biotech Ltd. Co. (Shanghai, China). Total RNA was used for removing the rRNAs with NEBNext® rRNA Depletion Kit (New England Biolabs, Inc., Massachusetts, USA) following the manufacturer's instructions.

RNA libraries were constructed by using rRNA-depleted RNAs with TruSeq Stranded Total RNA Library Prep Kit (Illumina, USA) according to the manufacturer’s instructions. Libraries were controlled for quality and quantified using the BioAnalyzer 2100 system (Agilent Technologies, USA). Library sequencing was performed on an illumina Novaseq 6000 instrument with 150 bp paired end reads. The RNA sequencing data were upload to GEO database (GSE228881, Token number: sfobcgaibvyrpgt).

### LncRNA–miRNA–mRNA network construction

LncRNA target genes were predicted by the locations to nearby genes. MiRmap, micorT, miRanda, PicTar and TargetScan were used to predict miRNA-mRNA target relationship. Cytoscape was used for visualization of the network.

### Correlation analysis

SPSS23 was used for correlation analysis between gene expression level and PANSS score, and partial correlation analysis was used for calculation after controlling age, sex and education level.

### GO and KEGG pathway analysis and PPI network construction

GO function (including biological process (BP), molecular function (MF) and KEGG pathway analysis [15–17] by bioinformations (http://www.bioinformatics.com.cn). GO and KEGG pathways with a *P*-value < 0.05 were supposed to be significantly enriched. Proteinprotein interaction (PPI) network was created by the Search Tool for the Retrieval of Interacting Genes database (STRING-Version 10.0, http://stringdb.org) with the interaction score > 0.4, and Cytoscape software was used to visualize and analyze the biological networks.

### ROC analysis

The diagnostic efficacy of lncRNA, miRNA, mRNA and lncRNA-miRNA-mRNA network were examined by receiver operating characteristic (ROC) analysis. ROC curves was performed using the OmicStudio tools at https://www.omicstudio.cn/tool/58.

### Statistical analysis

In demographic information, data were presented as mean ± standard deviation (SD). The significant differences were analyzed with SPSS version 23.0. Statistical analyses of age and education level which were normally distribution were performed using student’s *t*-test. Statistical analysis of gender were performed using Chi-square test. Differentially expressed lncRNA, miRNA and mRNA between two groups were filtered by Fold Change (Fold Chang ≥ 2, log(FC) >  = 1.0) and *P*-value (*P*-value ≤ 0.05). The significant differences were performed using student’s *t*-test.

## Results

### Demographic information of study subjects

We recruited 10 FOS and 10 matched HCs. The age, sex, and education level of 10 FOS and 10 HCs showed no significant differences. The mean value of PANSS score for FOS was 75.6 (Table 1).

**Table 1:** Demographics and clinical characters for HCs and FOS

	**FOS (*n* = 10)**	**HCs (*n* = 10)**	***P*-value**
Age (years)	34.2 ± 12.01	33.6 ± 12.05	0.9124
Males (%)	50	50	1.0000
Education level (years)	10.9 ± 3.665	9.9 ± 3.178	0.5227
PANSS total scores	75.60 ± 18.63		

### Identification of differentially expressed lncRNA, miRNA and mRNA in plasma exosome

**Fig. 2:**
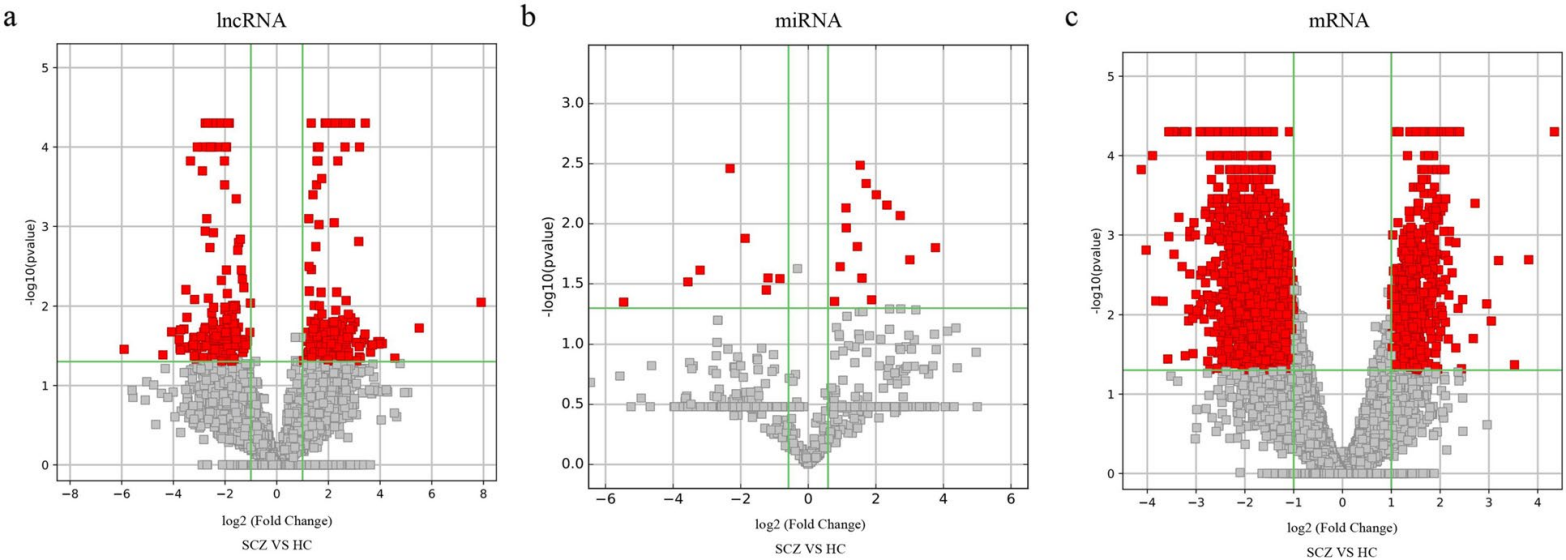
Volcano plots of exosomal DE-lncRNAs (**a**), DE-miRNAs (**b**) and DE-mRNAs (**c**) in SZ. The red points indicate differently expressed lncRNA, miRNA and mRNA

To identify differentially expressed exosomal circRNAs, miRNAs and mRNAs in exosomes in SZ, we performed RNA sequencing on plasma exosomes from 10 FOS patients and 10 HCs. There were 385 up-regulated and 361 down-regulated lncRNA, 14 up-regulated and 8 down-regulated miRNA, 690 up-regulated and 1947 down-regulated mRNA (Fig. 2, Supplementary Table S1).

### Construction lncRNA-miRNA and miRNA-mRNA network

**Fig. 3:**
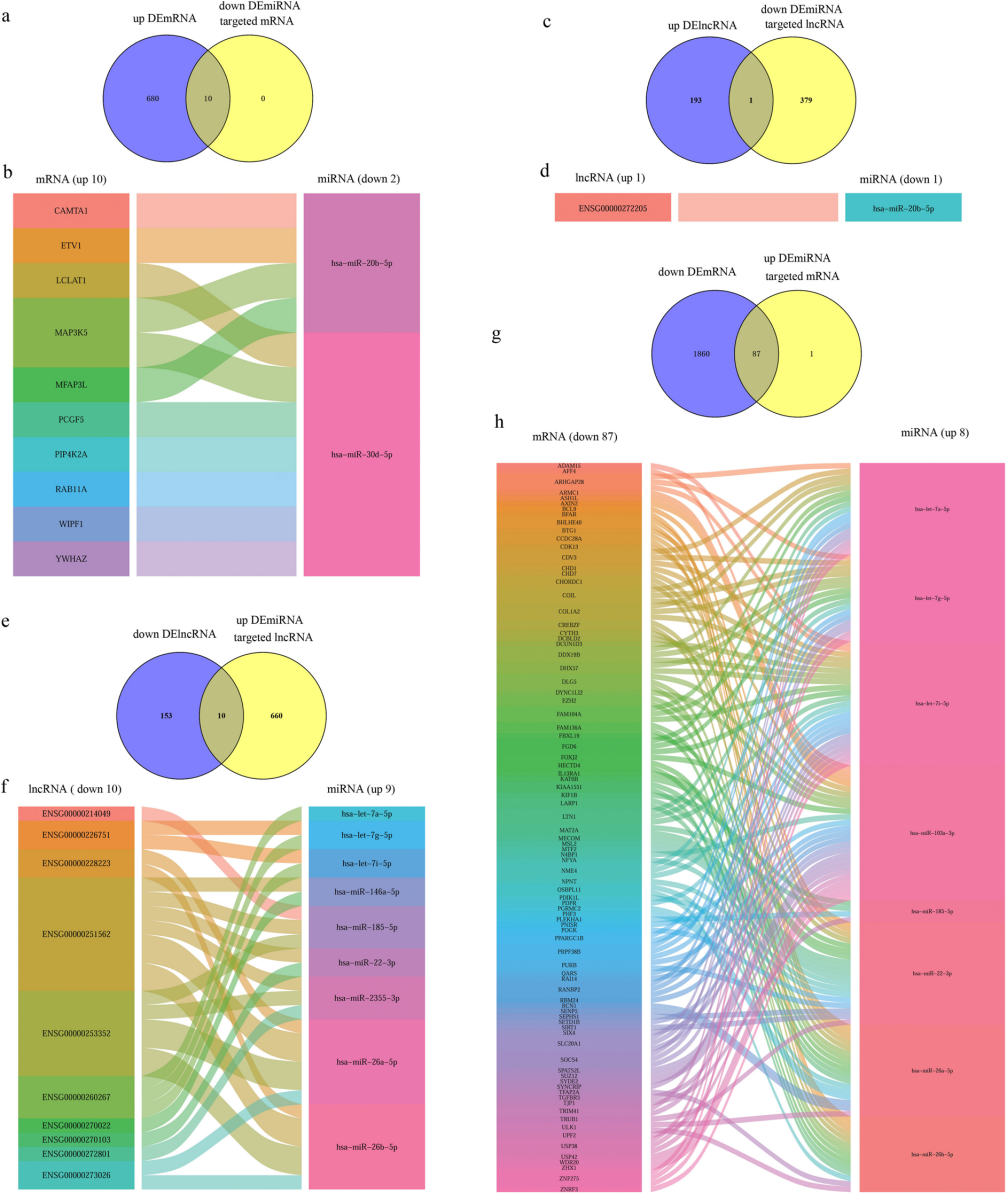
Construction of a lncRNA-miRNA and miRNA-mRNA network. **a** Venn diagram of overlapping mRNAs between the up-regulated mRNA and down-regulated miRNA targeted mRNA. **b** alluvial plot of miRNA(down-regulated)-mRNA(up-regulated). **c** Venn diagram of overlapping lncRNAs between the up-regulated lncRNA and down-regulated miRNA targeted lncRNA. **d** alluvial plot of lncRNA(up-regulated)-miRNA(down-regulated). **e** Venn diagram of overlapping lncRNAs between the down-regulated lncRNA and up-regulated miRNA targeted lncRNA. **f** alluvial plot of lncRNA(down-regulated)-miRNA(up-regulated). **g** Venn diagram of overlapping mRNAs between the down-regulated mRNA and up-regulated miRNA targeted mRNA. **h** alluvial plot of miRNA (up-regulated)-mRNA(down-regulated)

Then, we constructed lncRNA-miRNA and miRNA-mRNA network based on differentially expressed lncRNAs, miRNAs and mRNAs. Firstly, the target lncRNAs and mRNAs of down-regulated miRNA were identified. There are 380 down-regulated miRNA targeted lncRNA. The venn map was used to analyze the intersection between DEmiRNA target lncRNA and DElncRNA, and 1 up-regulated lncRNA (Fig. 3c) were identified. According to 1 up-regulated lncRNA, 1 down-regulated miRNA were recognized reversely (Fig. 3d). There were 10 overlapped up-regulated mRNA between down-regulated miRNA targeted mRNA and up-regulated mRNA (Fig. 3a). According to these 10 up-regulated mRNA, 2 down regulated mRNA were recognized (Fig. 3b).

Subsequently, the target lncRNAs and mRNAs of up-regulated miRNA were identified. There were 670 up-regulated miRNA targeted lncRNA, and 10 down-regulated lncRNA were identified in venn map (Fig. 3e). According to these 10 down-regulated lncRNA, 9 up-regulated miRNA were recognized (Fig. 3f). There are 88 up-regulated miRNA targeted mRNA, and 87 down-regulated mRNA were identified in venn map (Fig. 3g). According to these 87 down-regulated mRNA, 8 up-regulated miRNA were recognized (Fig. 3h).

### Construction lncRNA-miRNA-mRNA ceRNA network

**Fig. 4:**
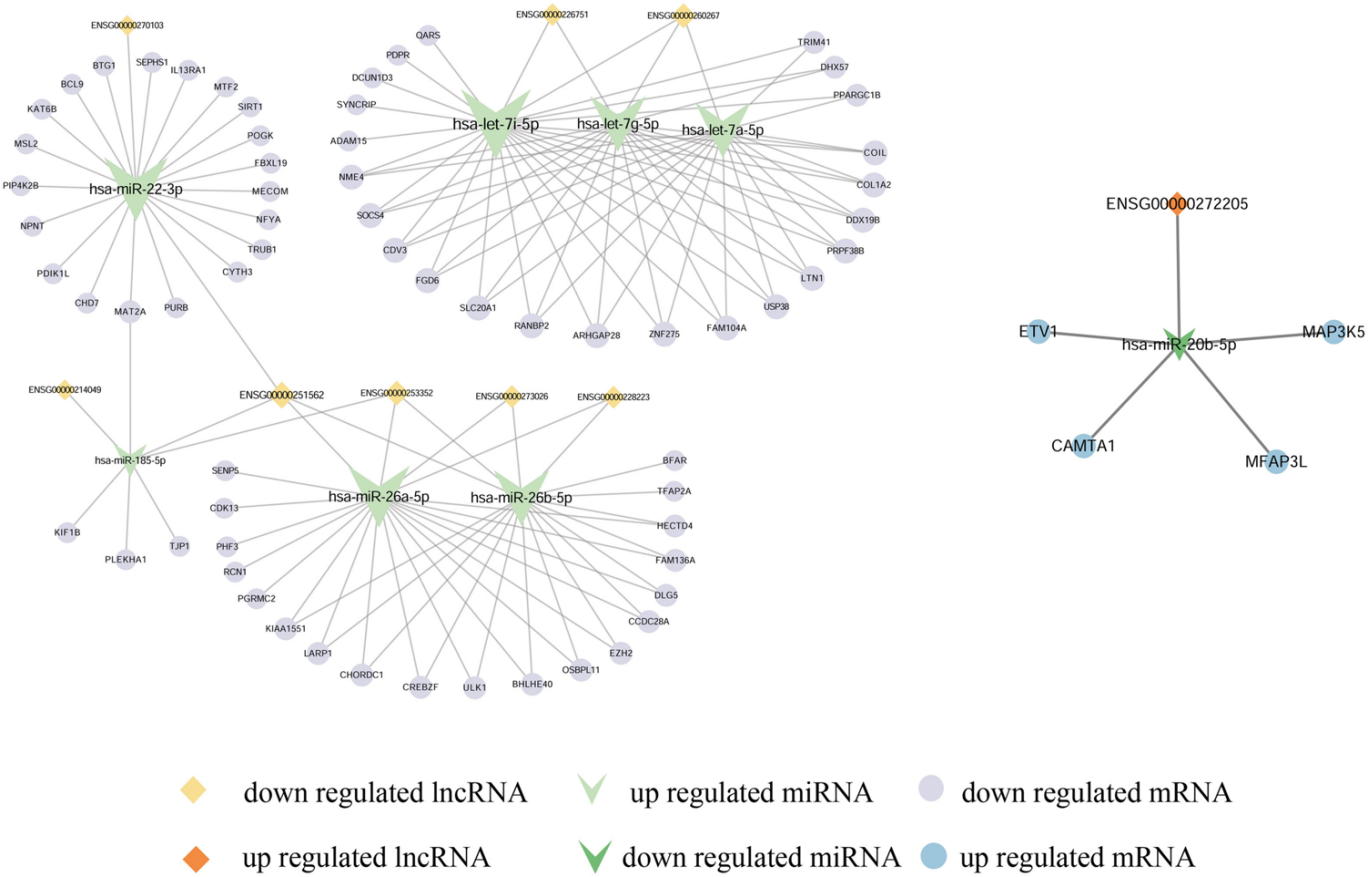
Construction of a lncRNA-miRNA-mRNA ceRNA network. lncRNA(down-regulated)-miRNA(up-regulated) -mRNA(down-regulated) regulatory network (left). The network consisting 8 lncRNA, 7 miRNA and 65 mRNA. lncRNA(up-regulated)-miRNA(down-regulated)-mRNA(up-regulated) regulatory network (right). The network consisting 1 lncRNA, 1 miRNA and 4 mRNA. The network was generated by Cytoscape. Down-regulated lncRNAs were represented by diamonds in light orange. Up-regulated lncRNAs were represented by diamonds in orange. Up-regulated miRNAs were represented by V-shape in light green. Down-regulated miRNAs were represented by V-shape in green. Down-regulated mRNAs were represented by circle in green purple. Up-regulated mRNAs were represented by circle in blue. Larger circles indicates more numbers of connections of genes

Then we constructed the ceRNA network. Taking the intersection of 9 up-regulated miRNA in Fig. 3f and 8 up-regulated miRNA in Fig. 3g, and found 7 overlapped up-regulated miRNA, combing the interacted lncRNA and mRNA, then the ceRNA network including 8 down-regulated lncRNA, 7 up-regulated miRNA and 65 down-regulated mRNA were constructed which including 129 edges (Fig. 4). In the same way, the ceRNA network including 1 up-regulated lncRNA, 1 down-regulated miRNA and 4 up-regulated mRNA were constructed which including 5 edges (Fig. 4).

### The gene expression level of exosomes ceRNA network correlated with the PANSS score

In order to explore the relationship between genes expression level in exosomal ceRNA network and clinical severity, the correlation between gene expression levels and PANSS scores including positive score, negative score, general psychophathology score and PANSS score (Sum of the first three values) was examined. In lncRNA, ENSG00000251562 was positively correlated with the negative score and PANSS score. None of miRNAs were associated with PANSS In mRNA, BFAR and HECTD4 were positively correlated with negative score, general psychophathology score and PANSS score. CHORDC1 and POGK were positively correlated with negative score and PANSS score. PRPF38B, ULK1 were positively correlated with negative score. TRUB1 was negatively correlated with negative score (Table 2).

**Table 2:** Association of lncRNA, miRNA and mRNAs in exosomal ceRNA network with PANSS score

		lncRNA						
		ENSG00000214049	ENSG00000228223	ENSG00000226751	ENSG00000253352	ENSG00000251562	ENSG00000260267	ENSG00000270103
Positvie score	Correlation Coefficient	-0.495	0.002	-0.18	-0.319	0.271	0.264	0.561
	*p*-value	0.259	0.997	0.7	0.486	0.556	0.567	0.19
Negative score	Correlation Coefficient	0.124	-0.021	-0.262	0.611	0.848	-0.164	-0.138
	*p*-value	0.792	0.964	0.571	0.145	0.016*	0.726	0.768
Psychopathology score	Correlation Coefficient	-0.455	0.006	-0.476	0.232	0.722	0.169	0.181
	*p*-value	0.305	0.99	0.28	0.616	0.067	0.717	0.698
PANSS score	Correlation Coefficient	-0.192	-0.079	-0.412	0.352	0.794	0.043	0.059
	*p*-value	0.68	0.866	0.359	0.438	0.033*	0.928	0.9

		lncRNA	miRNA	mRNA							
		ENSG00000272205	hsa-let-7a-5p	hsa-let-7g-5p	hsa-let-7i-5p	hsa-miR-185-5p	hsa-miR-22-3p	hsa-miR-26a-5p	hsa-miR-26b-5p	NFYA	ETV1
Positvie score	Correlation Coefficient	0.348	-0.466	-0.481	-0.496	-0.441	-0.105	-0.572	-0.169	0.698	0.448
	*p*-value	0.444	0.292	0.275	0.258	0.322	0.823	0.18	0.717	0.081	0.313
Negative score	Correlation Coefficient	-0.429	-0.274	-0.31	-0.304	-0.524	-0.166	-0.272	-0.321	0.19	-0.26
	*p*-value	0.337	0.552	0.499	0.508	0.228	0.723	0.556	0.482	0.683	0.573
Psychopathology score	Correlation Coefficient	-0.207	-0.45	-0.527	-0.423	-0.403	0.1	-0.578	-0.63	0.376	-0.2
	*p*-value	0.656	0.311	0.224	0.344	0.37	0.832	0.174	0.129	0.406	0.667
PANSS score	Correlation Coefficient	-0.179	-0.383	-0.469	-0.445	-0.519	-0.05	-0.498	-0.479	0.401	-0.126
	*p*-value	0.7	0.397	0.289	0.317	0.233	0.914	0.255	0.276	0.373	0.787

		mRNA														
		CYTH3	CCDC28A	FAM136A	RCN1	KIF1B	ZNF275	CDK13	MECOM	SEPHS1	ARHGAP28	PDPR	CDV3	SIRT1	FBXL19	NME4
Positvie score	Correlation Coefficient	-0.404	-0.117	0.029	-0.002	-0.126	-0.303	0.043	0.133	-0.051	0.693	0.005	-0.035	-0.506	0.005	-0.036
	*p*-value	0.369	0.803	0.951	0.997	0.788	0.509	0.927	0.776	0.914	0.084	0.992	0.94	0.247	0.992	0.938
Negative score	Correlation Coefficient	-0.179	0.372	-0.448	-0.471	-0.256	-0.258	0.538	-0.609	0.726	-0.31	0.444	0.534	0.35	-0.588	-0.463
	*p*-value	0.7	0.411	0.314	0.286	0.579	0.577	0.212	0.146	0.065	0.498	0.318	0.217	0.442	0.165	0.295
Psychopathology score	Correlation Coefficient	-0.426	0.112	-0.361	-0.32	-0.184	-0.511	0.269	-0.181	0.704	-0.037	0.302	0.071	-0.034	-0.319	-0.337
	*p*-value	0.341	0.811	0.426	0.485	0.693	0.241	0.559	0.697	0.078	0.938	0.51	0.879	0.942	0.485	0.46
PANSS score	Correlation Coefficient	-0.415	0.199	-0.345	-0.374	-0.253	-0.406	0.368	-0.379	0.626	-0.013	0.317	0.296	0.005	-0.419	-0.358
	*p*-value	0.354	0.669	0.448	0.409	0.585	0.366	0.417	0.401	0.133	0.978	0.489	0.519	0.992	0.349	0.431

		mRNA														
		BFAR	TJP1	EZH2	PLEKHA1	CHORDC1	BCL9	PHF3	SENP5	COIL	IL13RA1	FAM104A	BTG1	BHLHE40	PRPF38B	SYNCRIP
Positvie score	Correlation Coefficient	0.197	0.181	-0.264	0.047	0.476	-0.529	0.218	-0.004	0.044	-0.017	0.245	0.306	-0.071	0.207	-0.286
	*p*-value	0.672	0.697	0.568	0.92	0.28	0.222	0.638	0.993	0.926	0.972	0.597	0.504	0.88	0.656	0.534
Negative score	Correlation Coefficient	0.811	-0.504	-0.096	-0.376	0.762	-0.308	0.059	0.49	-0.467	0.013	-0.014	-0.113	0.217	0.809	-0.257
	*p*-value	0.027*	0.249	0.837	0.406	0.047*	0.502	0.9	0.264	0.29	0.977	0.977	0.809	0.64	0.028*	0.577
Psychopathology score	Correlation Coefficient	0.776	-0.329	-0.47	-0.435	0.741	-0.616	0.145	0.166	-0.262	-0.067	-0.181	0.024	0.198	0.663	-0.358
	*p*-value	0.04*	0.471	0.287	0.33	0.057	0.141	0.756	0.723	0.571	0.887	0.697	0.959	0.67	0.104	0.431
PANSS score	Correlation Coefficient	0.761	-0.37	-0.228	-0.36	0.808	-0.57	0.121	0.403	-0.36	0.031	-0.006	0.025	0.131	0.75	-0.354
	*p*-value	0.047*	0.414	0.623	0.428	0.028*	0.182	0.797	0.37	0.428	0.948	0.989	0.958	0.78	0.052	0.436

		mRNA														
		TFAP2A	CREBZF	PIP4K2B	MTF2	POGK	ADAM15	SLC20A1	OSBPL11	TRIM41	PURB	DLG5	RANBP2	LARP1	PPARGC1B	KAT6B
Positvie score	Correlation Coefficient	-0.457	-0.227	-0.44	0.17	0.287	0.044	0.136	-0.008	0.295	0.702	-0.649	0.144	0.413	-0.076	0.45
	*p*-value	0.303	0.625	0.324	0.715	0.532	0.926	0.771	0.987	0.52	0.079	0.114	0.758	0.357	0.871	0.312
Negative score	Correlation Coefficient	0.072	0.406	-0.302	0.119	0.871	-0.174	0.509	0.401	-0.377	0.16	-0.007	0.041	-0.402	-0.323	0.079
	*p*-value	0.878	0.366	0.51	0.799	0.011*	0.709	0.243	0.372	0.405	0.733	0.988	0.93	0.371	0.48	0.866
Psychopathology score	Correlation Coefficient	-0.307	-0.06	-0.68	0.01	0.641	0.022	0.249	0.164	-0.103	0.355	-0.401	0.014	-0.207	-0.212	0.188
	*p*-value	0.504	0.899	0.093	0.984	0.121	0.963	0.59	0.725	0.825	0.435	0.372	0.977	0.656	0.649	0.686
PANSS score	Correlation Coefficient	-0.246	0.151	-0.541	0.081	0.763	-0.079	0.393	0.246	-0.192	0.416	-0.299	0.066	-0.172	-0.293	0.238
	*p*-value	0.594	0.746	0.21	0.863	0.046*	0.867	0.383	0.595	0.68	0.353	0.514	0.888	0.712	0.524	0.607

		mRNA														
		DDX19B	DHX57	PGRMC2	COL1A2	TRUB1	NPNT	MAT2A	USP38	CHD7	CAMTA1	QARS	HECTD4	MSL2	KIAA1551	PDIK1L
Positvie score	Correlation Coefficient	0.371	0.257	-0.137	-0.182	0.253	0.242	0.259	-0.036	-0.077	-0.275	-0.459	0.473	0.311	0.427	-0.4
	*p*-value	0.412	0.578	0.77	0.696	0.585	0.601	0.575	0.939	0.87	0.551	0.3	0.284	0.497	0.339	0.373
Negative score	Correlation Coefficient	0.032	-0.079	-0.089	-0.591	-0.836	0.618	0.609	0.548	-0.485	-0.055	-0.096	0.815	0.59	0.401	-0.019
	*p*-value	0.945	0.867	0.85	0.163	0.019*	0.139	0.147	0.203	0.27	0.907	0.838	0.025*	0.163	0.373	0.968
Psychopathology score	Correlation Coefficient	0.261	0.084	-0.187	-0.636	-0.363	0.598	0.578	0.29	-0.305	-0.238	-0.231	0.858	0.601	0.338	-0.149
	*p*-value	0.572	0.858	0.688	0.125	0.423	0.156	0.174	0.527	0.506	0.607	0.619	0.014*	0.154	0.459	0.75
PANSS score	Correlation Coefficient	0.225	-0.005	-0.153	-0.602	-0.524	0.59	0.62	0.411	-0.338	-0.121	-0.26	0.868	0.632	0.464	-0.222
	*p*-value	0.628	0.992	0.744	0.152	0.227	0.163	0.138	0.36	0.458	0.796	0.573	0.011*	0.128	0.294	0.632

		mRNA						
		ULK1	SOCS4	FGD6	DCUN1D3	MAP3K5	LTN1	MFAP3L
Positvie score	Correlation Coefficient	0.006	-0.17	0.302	0.457	-0.158	0.526	-0.239
	*p*-value	0.99	0.715	0.511	0.303	0.735	0.225	0.605
Negative score	Correlation Coefficient	0.791	0.204	-0.042	-0.588	-0.305	0.199	0.059
	*p*-value	0.034*	0.66	0.928	0.165	0.506	0.669	0.9
Psychopathology score	Correlation Coefficient	0.744	0.006	0.013	-0.149	-0.146	0.306	-0.116
	*p*-value	0.055	0.99	0.978	0.75	0.754	0.505	0.804
PANSS score	Correlation Coefficient	0.713	0.072	0.036	-0.269	-0.237	0.344	-0.002
	*p*-value	0.072	0.878	0.939	0.559	0.609	0.45	0.996

* p-value<0.05

### GO and KEGG pathway analysis of DemRNA in ceRNA network

**Fig. 5:**
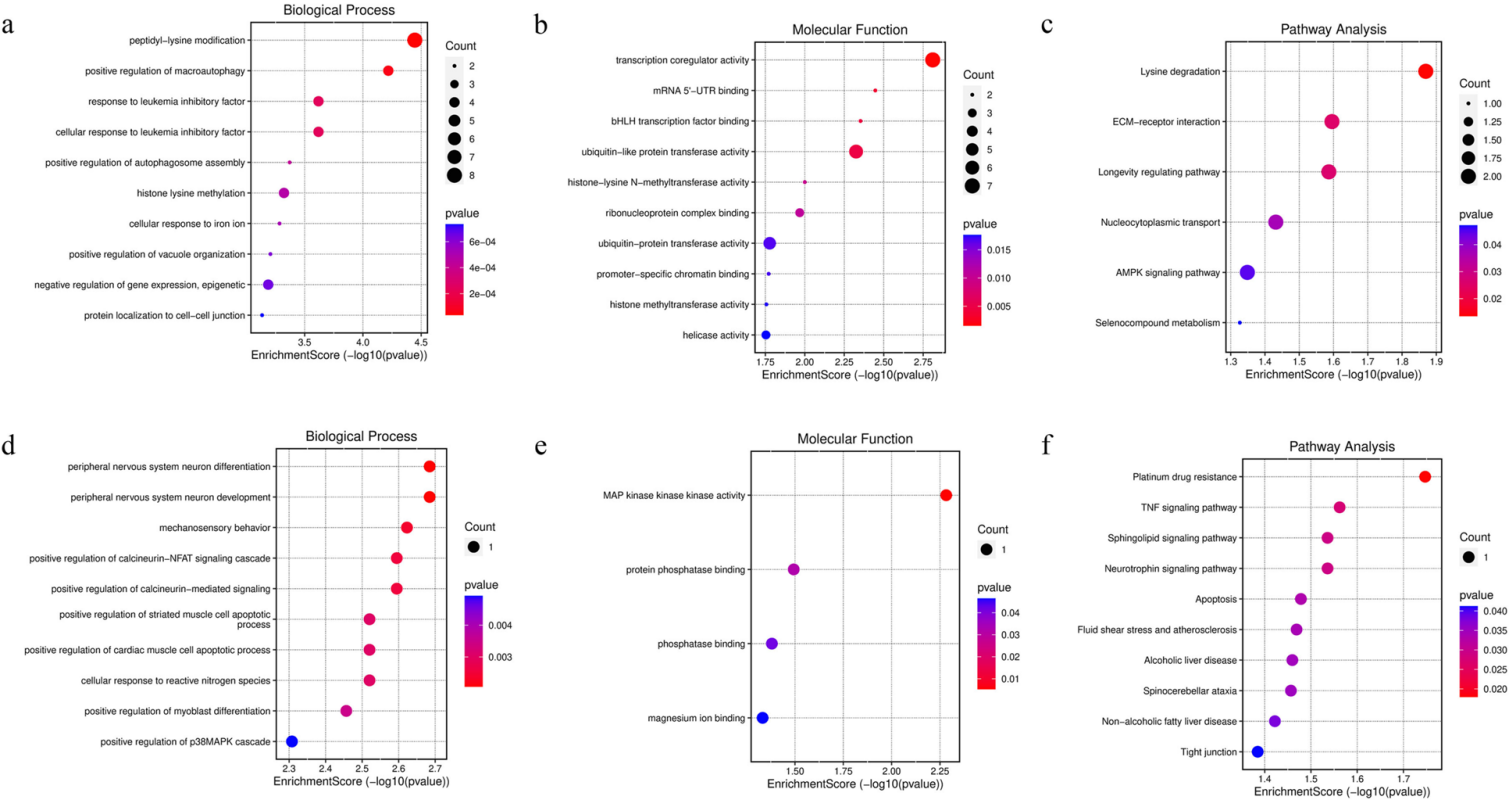
Go functional annotation and KEGG pathway enrichment analysis for mRNA. Bubble plot of BP (**a**), MF (**b**) and KEGG pathway analysis (**c**) of down-regulated mRNAs in lncRNA-miRNA-mRNA. Bubble plot of BP (**d**), MF (**e**) and KEGG pathway analysis (**f**) of up-regulated mRNAs in lncRNA-miRNA-mRNA. From blue to red, the enrichment increases. Larger circles indicates more significant proportion of genes

In order to explore the potential mechanisms of mRNA enriched in CeRNA network, GO and pathway enrichment analysis were performed. The down DEmRNA in BP were enriched in peptidyl-lysine modification, positive regulation of macroautophagy, response to leukemia inhibitory, cellular response to leukemia inhibitory factor, cellular response to leukemia inhibitory factor (Fig. 5a). The down regulated mRNA in MF were enriched in transcription coregulator activity, mRNA 5’-UTR binding, bHLH transcription factor binding, ubiquitin-like protein transferase activity, histone-lysine N-methyltransferase activity (Fig. 5b). As for the the KEGG analysis, the down-regulated mRNA were enriched in lysine degradation, ECM-receptor interacton, longevity regulating pathway, nucleocytoplasmic transport, AMPK signaling pathway, selenocompound metabolism (Fig. 5c).

The BP terms of up-regulated mRNA were enriched in peripheral nervous system neuron differentiation, peripheral nervous system neuron development, mechanosensory behavior, positive regulation of calcineurin-NFAT signaling cascade, positive regulation of calcineurin-mediated signaling (Fig. 5d). The MF terms of up-regulated mRNA were enriched in MAP kinase kinase kinase activity, protein phosphatase binding, phosphatase binding and magnesium ion binding (Fig. 5e). By the KEGG analysis, the up-regulated mRNA were enriched in platinum drug resistance, TNF signaling pathway, sphingolipid signaling pathway, apoptosis (Fig. 5f)

### PPI network construction and hub genes identification

**Fig. 6:**
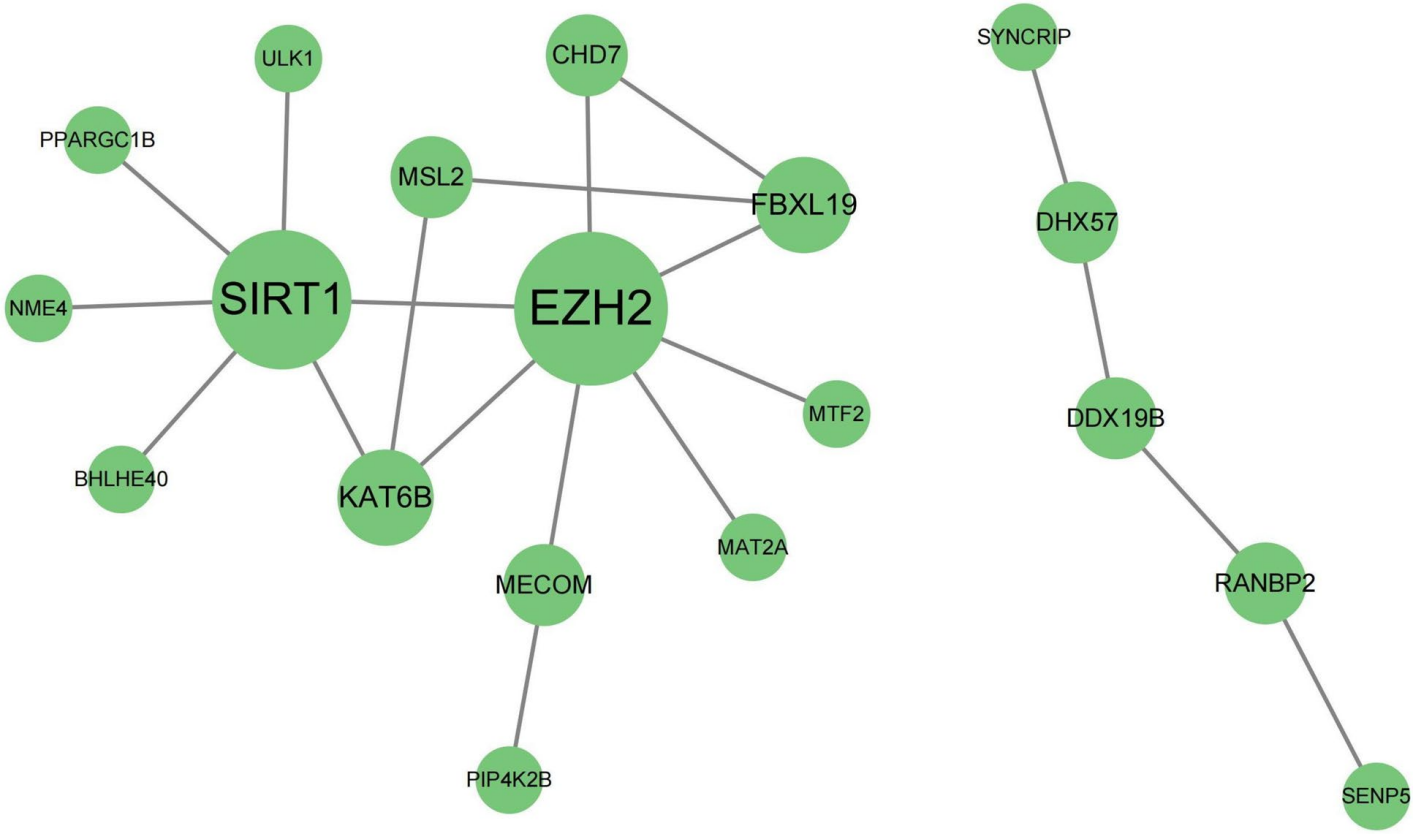
PPI network of down-regulated mRNAs in lncRNA-miRNA-mRNA. The size of the node represents the degree

In order to find out the functional association between the identified genes, the PPI network was constructed based on the STRING online database and visualized by Cytoscape software. The PPI network contained 19 nodes and 20 edges. According to the number of connection indicated from Cytoscape’ s cytoHubba plugin, the EZH2(7) and SIRT1(6) were the identified as hub gene (Fig. 6), and were used in subsequently study.

### Receiver operating characteristic analysis of lncRNA, miRNA and mRNA

**Fig. 7:**
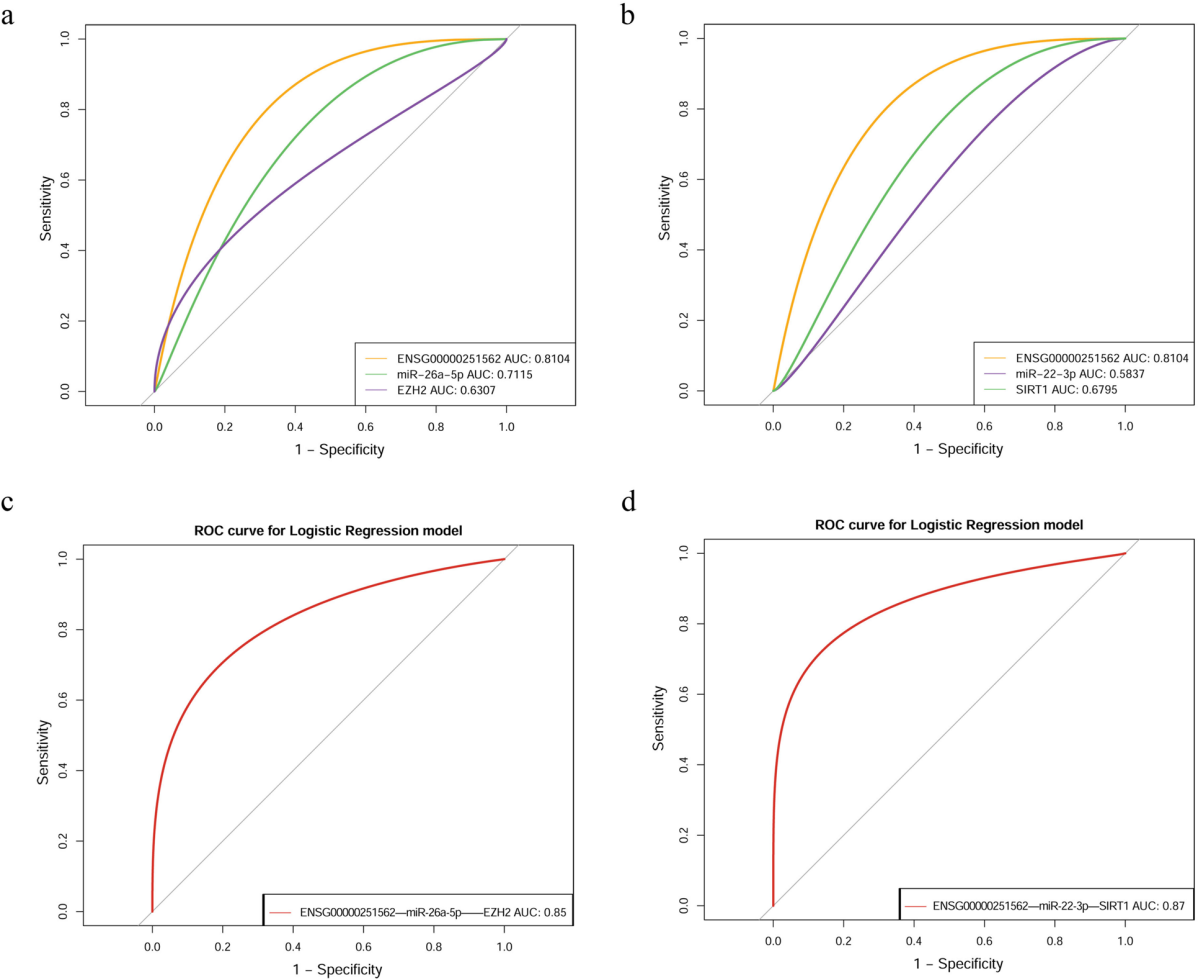
ROC curves of lncRNA, miRNA, mRNA and lncRNA-miRNA-mRNA network. **a** ENSG00000251562, miR-26a-5p, EZH2. **b** ENSG00000251562, miR-22-3p, SIRT1. **c** ENSG00000251562—miR-26a-5p—EZH2. **d** ENSG00000251562—miR-22-3p—SIRT1. ROC analysis expressed as area under the curve (AUC)

In order to examine the diagnostic performances of lncRNA, miRNA, mRNA and lncRNA-miRNA-mRNA network in ceRNA, receiver operating characteristic (ROC) analysis were performed. Down-regulated mRNA, EZH2 and SIRT1 which were the identified as hub gene in 3.7, were selected. In lncRNA-miRNA-mRNA ceRNA network, there are multiple upstream miRNA and lncRNA of EZH2 and SIRT1. MiRNAs and lncRNAs associated with neuropsychiatric diseases were selected for ROC analysis (ENSG00000251562, miR-26a-5p) (Table 3). ROC analysis showed an the area under the ROC curve (AUC) of 0.8104 for ENSG00000251562, 0.7115 for miR-26a-5p, 0.6307 for EZH2, 0.5837 for miR-22-3p, 0.6795 for SIRT1. The AUC of ENSG00000251562—miR-26a-5p—EZH2 and ENSG00000251562—miR-22-3p—SIRT1 were 0.85 and 0.87, respectively (Fig. 7). The AUC was found to be highest for lncRNA-miRNA-mRNA network than lncRNA, miRNA, mRNA alone.

**Table 3:** lncRNA and miRNA related diseases

Type	Gene symbol	Related diseases	References
lncRNA	ENSG00000251562	bipolar disorder	[18]
	ENSG00000253352	Cervical cancer, Prostate cancer, Bladder Cancer	[19–21]
	ENSG00000273026	Not reported	
	ENSG00000228223	Not reported	
	ENSG00000270103	Not reported	
miRNA	miR-26a-5p	Parkinson's disease Alzheimer's disease	[22, 23]
	miR-26b-5p	Bladder cancer, Pulmonary cancer, Multiple myeloma	[24–26]

## Discussion

The study of biomarkers for SZ has attracted much attention, and exosomes have shown potential as biomarkers in a variety of diseases. Exosome-derived circRNAs, miRNAs, proteins, and metabolites have been reported in SZ. It’s reported there are 44 differentially expressed exo-circRNAs in SZ patients’ and HCs’ plasma, which played roles in pathogenesis regarding metabolic process, stress response, and histone ubiquitination [27]. Du et al. performed miRNA expression profiling in serum-derived exosome from first-episode SZ and HCs, and found 11 miRNAs in serum exosomes which can be used to classify samples from SZ patients and HCs and could be promising biomarkers for SZ [14]. Rnganathan et al. examined the neuropathology relevant protein in exosomes from SZ patients and found that GFAP significantly increased and α-II-spectrin significantly decreased in SZ patients compared with HCs [28]. Du et al. identified 25 metabolites in SZ patients which can be used to classify samples from SZ patients and HCs [29]. However, exosomal lncRNA, and exosomal lncRNA-miRNA-mRNA network have not reported yet. In this study, we reported that there are 746 differently expressed lncRNA, 22 differently expressed miRNA, and 2637 differently expressed mRNA (Fig. 2), and performed the ceRNA network analysis.

CeRNAs in exosomes have also been reported, such as malignant tumors [30], endometriosis [31], and ventricular septal defect [32], exosomal ceRNAs play physiological roles in these diseases and have the potential to serve as biomarkers. In this study, we construct exosomal lncRNA-miRNA-mRNA network based on the interaction between different expressed lncRNA, miRNA and mRNA, which included 8 down-regulated lncRNA, 7 up-regulated miRNA and 65 down-regulated mRNA and 129 edges (Fig. 4), and 1 up-regulated lncRNA, 1 down-regulated miRNA, 4 up-regulated mRNA and 5 edges (Fig. 4). In the ceRNA network, ENSG00000251562, BFAR, HECTD4, CHORDC1, POGK, PRPF38B and ULK1was positively correlated with PANSS score. TRUB1 was negatively correlated with negative score (Table 2). It is reported that higher astrocyte-derived exosomal P-T181-tau levels were associated with worse executive functioning in SZ [33], implying exosomal genes expression levels were associated with clinical severity. However, none of miRNAs were associated with PANSS (Table 2), this may due to the relatively small sample size (10 patients). In future studies, we will expand the sample size and further examine the correlation between miRNA expression level and PANSS score. Then we explore the GO and KEGG pathway of mRNA in network, and found that the up-regulated mRNA enriched in peripheral nervous system neuron differentiation, peripheral nervous system neuron development in BP (Fig. 5d). However, the down-regulated mRNA didn’t enriched in neuropsychiatric related pathway in MF, BP and KEGG pathway (Fig. 5a-c).

To better analyze the 65 down-regulated mRNAs, we performed PPI network analysis and identified two hub genes, EZH2 and SIRT1 (Fig. 6). Subsequently, we performed ROC analysis on these two genes and their ceRNA.

Enhancer of zeste homolog 2 (EZH2) plays important roles in development and function of central nervous system (CNS). The timely expression of EZH2 maintained a balance of neural stem cell (NSC) self-renewal and differentiation, whereas removal of EZH2 inhibits NSC proliferation. Furthermore, EZH2 regulated NSC fate determination, in which enhanced EZH2 drives NSC progression toward neuronal and oligodendrocyte lineages at the expense of astrocytes [34]. The upstream miRNA of EZH2 was miR-26a-5p and miR-26b-5p. Mir-26a-5p has been identified as a miRNA associated with inflammation in a variety of pathological processes. Luarte et al. reported that miR-26a-5p is involved in the process of astrocyt-derived small extracellular vesicles regulating dendritic complexity [35]. Mir-26a-5p plays important roles in Parkinson's disease and Alzheimer's disease [22, 23]. At present, most of the reports on miR-26b-5p are concentrated in the field of cancer [24–26], and there is no report in the field of psychiatry. So we selected the miR-26a-5p for ROC curve detection. The four upstream lncRNA of miR-26a-5p: ENSG00000251562, ENSG00000253352, ENSG00000273026 and ENSG00000228223. ENSG00000251562 was reported involved in bipolar disorder [18]. ENSG00000253352 was reported involved in cervical cancer [19], prostate cancer [20] and Bladder Cancer [21]. No disease have been reported for ENSG00000273026 ENSG00000228223. In additon, the expression level of ENSG00000251562 was positively correlated with the negative score and PANSS score (Table 2), so ENSG00000251562 was selected to perform the ROC curve detection. Our results showed that the EZH2 was down-regulated, miR-26a-5p was up-regulated, ENSG00000251562 was down-regulated in plasma exosomal derived from SZ (Fig. 4), implying that the exosomes play important role in proliferation, growth and differentiation of CNS [36, 37].

Sirtuin (SIRT1) plays essential role in regulating cell survival, apoptosis, inhibiting the stress-induced inflammatory response [38], regulating biological rhythm and transducing dopaminergic signals [39]. Wang et al. repored that SIRT1 gene is associated with SZ in Eastern Asian populations, especially in Japanese and Chinese Han populations [40]. Low plasma SIRT1 mRNA levels are associated with depressive symptoms in SZ patients [41]. subsequently, Wang et al. reported that SIRT1 mRNA level was correlation with rs3758391, a SNP in SIRT1, and rs3758391 is a risk factor for SZ pathogenesis,especially associated with depressive symptoms [40]. Lee et al. reported that after SIRT1 was inhibited in mice with the inhibitor nicotinamide, mice developed a phenotype of dopamine deficiency, small size, weight loss, and decreased motor activity, suggesting an effect of SIRT1 on the dopaminergic system [42]. The upstream miRNA of SIRT1 has been reported in SZ. Saud et al. reported that miR-22-3p up-regulated in the blood of SZ patients compared with HC [43]. JieMa et al. reported that miR-22-3p, miR-92A-3p and miR-137 found could be used in combination as biomarkers of SZ, and the target genes of mir-22-3p, mir-92a-3p and mir-137 are related to synaptic structure and function [44]. The upstream lncRNA of miR-22-3p ENSG00000270103 has not been reported in psychiatric disorders. So the ENSG00000251562 was selected as the upstream of miR-22-3p in subsequently analysis. In this study, SIRT1 was down-regulated, miR-22-3p was up-regulated, ENSG00000251562 was down-regulated in plasma exosomal derived from SZ (Fig. 4), implying that exosomes may participate in dopaminergic signaling pathway and thus participate in the regulation of SZ.

ROC analysis was performed on the above lncRNAs, miRNAs and mRNAs and their networks, and found that the AUC of lncRNA-miRNA-mRNA network was higher than lncRNA, miRNA and mRNA alone (Fig. 7). Liu et al. reported that The EGR1—miR-30a-5P—NEUROD1 axis possessed greater diagnostic value than miR-30a-5p alone [45], suggesting that multimolecular networks are better candidates for diagnostic markers than individual molecules.

Exosomes, characterized by their ability to cross the blood–brain barrier, can be used as drug delivery carriers to enter the brain. Curcumin-primed exosomes could prevent the neuronal death to relieve the symptoms of Alzheimer's disease by inhibiting phosphorylation of the Tau protein through activating the AKT/GSK-3β pathway [46]. Haney et al. have developed a novel exosomal-based delivery system for a potent antioxidant catalase for the treatment of Parkinson's disease (PD). After intranasal administration, catalase-loaded exosome (exoCAT) can accumulate significantly in the brain of PD mouse and play a significant neuroprotective role in both in vivo and in vitro models of PD [47]. Engineered extracellular vesicle carrying circDYM can be delivered to the brain, improved depression-like behavior in mouse models of chronic unpredictable stress (CUS) and inhibiting neuroinflammatory signaling and microglial activation [48]. Exosomes as a drug delivery system for the treatment of SZ has not been reported. The role of exosome lncRNA-miRNA-mRNA ceRNA network in SZ and whether the engineered exosomes can alleviate or prevent the symptoms of schizophrenia are a promising subject, and we will continue to pay attention to this area in future studies.

## Conclusion

We constructed lncRNA-miRNA-mRNA ceRNA network in plasma exosomes derived from FOS patients and HCs, and examined the diagnostic performances found that lncRNA-miRNA-mRNA network had the potential to be used as a biomarker for FOS. In future studies, we will expand the sample size to further verify the expression of genes in ceRNA, the binding and regulatory effects of lncRNA on miRNA and miRNA on mRNA in ceRNA network also need to be further verified.

### Supplementary Information

**Additional file 1.**

## Data Availability

The datasets used and/or analyzed during the current study were upload to GEO database (GSE228881).
